# Risk of Developing Dementia at Older Ages in the United States

**DOI:** 10.1007/s13524-017-0598-7

**Published:** 2017-08-03

**Authors:** Ezra Fishman

**Affiliations:** HealthCore, Inc., Wilmington, DE USA

**Keywords:** Cognition, Competing risks, Differential mortality, Health and Retirement Study (HRS), Alzheimer’s disease

## Abstract

Dementia is increasingly recognized as a major source of disease burden in the United States, yet little research has evaluated the lifecycle implications of dementia. To address this research gap, this article uses the Aging, Demographics, and Memory Study (ADAMS) to provide the first nationally representative, longitudinal estimates of the probability that a dementia-free person will develop dementia later in life. For the 1920 birth cohort, the average dementia-free 70-year-old male had an estimated 26.9 % (SE = 3.2 %) probability of developing dementia, and the average dementia-free 70-year-old female had an estimated 34.7 % (SE = 3.7 %) probability. These estimates of risk of dementia are higher for younger, lower-mortality cohorts and are substantially higher than those found in local epidemiological studies in the United States, suggesting a widespread need to prepare for a life stage with dementia.

**Electronic supplementary material:**

The online version of this article (doi:10.1007/s13524-017-0598-7) contains supplementary material, which is available to authorized users.

## Introduction

Dementia is increasingly recognized as a major source of disease burden in the United States and other developed countries (Murray et al. 2012). A recent study estimated that 8.8 % of adults aged 65 and older in the United States had dementia in 2012 (Langa et al. 2017), corresponding to approximately 3.65 million people. Dementia imposed a financial cost of approximately $28,500 per affected person per year, after accounting for comorbidities and not counting the economic cost of informal care (Hurd et al. 2013). As the U.S. population ages, the number of Americans with dementia is very likely to increase in the coming decades (He and Larsen 2014; Kasper et al. 2015).

This study has two aims. The first is to estimate the probability that an average dementia-free 70-year-old in the United States will develop dementia in his/her remaining life, and to describe the related quantities of dementia-free life expectancy and life expectancy with dementia. These quantities have been estimated using data from local epidemiological studies (Seshadri and Wolf 2007; Seshadri et al. 1997; Tom et al. 2015), but this study extends the literature by using nationally representative data with clinically validated diagnoses. The second aim of this article is to assess the extent to which long-run declines in adult mortality and possible delays or declines in dementia incidence affect the risk of developing dementia, the first such assessment in the literature.

Estimating the probability that an average dementia-free person of a given age will develop dementia in the course of his or her remaining life requires incorporating the competing risks of dementia onset and death. Such data on incidence and mortality also permit estimating the related measures of dementia-free life expectancy and life expectancy with dementia. These measures translate population-level quantities, such as incidence and relative risk of death, to the level of the individual. Such measures are therefore valuable for individuals, firms, and governments as they plan for retirement, save and contribute to pensions, and assess future health care costs and caregiving needs. Specifically, many people with dementia require assistance with activities of daily living (ADLs), often in residential facilities. For individuals with sufficiently low income and assets, Medicaid covers the costs of such long-term services and supports. Therefore, when drawn from nationally representative samples, these measures of risk of developing dementia can contribute to estimates of future Medicaid enrollment and costs. Dementia incidence has been studied at the national level (Plassman et al. 2011), but the incorporation of the competing risk of death without dementia has not. For demographers and epidemiologists, these quantities provide meaningful insight into the question of whether long-run gains in survivorship are being experienced in healthy or unhealthy states (Crimmins and Beltrán-Sánchez 2010; Crimmins et al. 2009).

## Data, Measures, and Methods

### Sample and Definitions

According to the Diagnostic and Statistical Manuals of Mental Disorders (DSM-IV), the essential feature of dementia is the development of multiple cognitive deficits that include memory impairment and at least one of the following: aphasia (language deficit), apraxia (movement deficit), agnosia (deficit in recognition of objects or senses), or executive functioning deficit (American Psychiatric Association 2000). The cognitive deficits must represent a decline from past abilities and must be severe enough to cause impairment in occupational or social functioning (American Psychiatric Association 2000). The most common type of dementia is Alzheimer’s disease (AD), which accounts for 60 % to 80 % of dementia cases; the next most common type is vascular dementia, which alone accounts for approximately 10 % of cases but often accompanies AD (Alzheimer’s Association 2014). The estimated average age of onset of dementia in the United States is 83.7 years old (Plassman et al. 2011), and dementia is often accompanied by comorbidities, such as diabetes and a history of stroke (Langa et al. 2017). Dementia on its own is associated with significantly increased risk of death, and comorbidities such as diabetes have been shown to increase the risk of death further among those with dementia (Todd et al. 2013).

This study uses the Aging, Demographics, and Memory Study (ADAMS), a nationally representative, longitudinal study of cognitive health and dementia conducted in four waves from 2001 to 2009 (Langa et al. 2005). ADAMS, a probability subsample of the Health and Retirement Study (HRS), examined adults aged 70 and older with a series of cognitive, psychological, and neurological tests, and conducted an extensive medical history, an inventory of current prescription medications, a neurology-focused physical exam, and a family/caregiver questionnaire. The testing was conducted in person by trained technicians and nurses and was supervised by neuropsychologists (Langa et al. 2005). Diagnostic criteria were based on the DSM-III-R and the DSM-IV, and a consensus expert panel of physicians made the final diagnosis of dementia (Heeringa et al. 2009; Langa et al. 2005). The clinical diagnoses in ADAMS are especially valuable because unlike for certain other chronic diseases, such as diabetes, there is no single test or measure that can provide a “gold standard” ascertainment of dementia status in large samples (Lees et al. 2014).

Detailed descriptions of the ADAMS sample have been previously published (Heeringa et al. 2009; Langa et al. 2005; Plassman et al. 2007). Briefly, a stratified random subsample of 1,770 subjects in the Health and Retirement Study (HRS) were targeted for inclusion in ADAMS; of these, 227 died before they could be assessed, 687 refused or did not participate in ADAMS for other reasons, and 856 participated in ADAMS. The ADAMS subjects did not differ significantly from study nonparticipants in terms of age, sex, or education, but they were more likely to have scored in the cognitively normal range on cognitive screening tests in earlier waves of HRS (Langa et al. 2005). All ADAMS sampling weights incorporate statistical adjustment for differences in HRS cognitive scores between respondents and nonrespondents (Heeringa et al. 2009).

The initial wave of ADAMS, 2001–2003, examined 856 subjects to generate baseline estimates of dementia prevalence in the United States (Plassman et al. 2007). The subsequent waves followed 456 dementia-free individuals for dementia incidence (Plassman et al. 2011); longitudinal sampling weights adjust for differential attrition by baseline cognitive status (Heeringa et al. 2009). The second wave focused on subjects whose baseline status was *cognitively impaired, no dementia* (CIND); this second wave assessed subjects 16 to 18 months after their baseline assessment. For the third and fourth waves, all living subjects who were dementia-free at baseline were in the sampling frame. Subjects in the third wave averaged 3.7 years since their most recent assessment, and subjects in the fourth wave averaged 1.8 years since their most recent assessment (Plassman et al. 2011). Despite the relatively long intervals between assessments, especially between Waves A and C for those not assessed in Wave B, ADAMS investigators could determine—based on informant reports, medical records, and clinical assessment—whether a subject experienced the onset of dementia at any time since the previous assessment. For example, if a 72-year-old subject was deemed dementia-free at baseline and then was assessed at age 76 and found to have dementia, investigators could determine that his age at the onset of dementia was 73. The assignment of ages at dementia onset during the interassessment interval allows for the estimation of dementia incidence rates rather than probabilities. Thus, the ADAMS data can be used to calculate age-specific incidence of dementia, an essential ingredient in making estimates of age-specific risk of developing dementia.

Mortality data come from ADAMS’ link to the HRS mortality tracking via the National Death Index (NDI), which provides vital status and, if deceased, month of death as of December 2011. The 856 ADAMS subjects constitute the individuals at risk of mortality. The ADAMS study team did not attempt to diagnose dementia posthumously in subjects who had not received a dementia diagnosis during their lifetimes. This study uses the mortality data to generate estimates of the age-specific ratio of mortality rates between those with and those without dementia. These estimated ratios reflect the distribution of comorbid conditions in the ADAMS subjects. They are thus nationally representative after the application of sampling weights, but they would not apply to future U.S. cohorts if the distribution of comorbidities among people with dementia changes. Mortality rates for the entire U.S. population come from the Social Security Administration (SSA) cohort life tables (Bell and Miller 2005).

### Demographic Methods

The quantities estimated from the data are age-specific rates of dementia onset (incidence) and age-specific mortality rates by dementia status (with dementia vs. without). After age-specific incidence rates and mortality rates by dementia status have been estimated, multiple-decrement life table relations will be used to track exits from the dementia-free population via death or dementia onset (Preston et al. 2001: chapter 4). The number of exits via dementia onset features prominently in the estimates of risk of developing dementia.

The primary quantity of interest is the probability of developing dementia for a dementia-free person age *a* (an age chosen by the investigator):

1 $$ {\Pi}_a=\frac{\left({\sum}_{x=a}^w{}_1{}^{dem}d_x^{DF}\right)}{l_a^{DF}}, $$

where Π_*a*_ is the probability that a dementia-free person age *a* will develop dementia before death; *w* is the highest age interval; *x* indexes each age, at and above age *a*; ${}_1{}^{dem}d_x^{DF}$ counts new cases of dementia (left superscripted *dem*) occurring in the previously dementia-free (right superscripted *DF*) population; the left subscripted 1 indicates that new cases of dementia are counted in single-year age intervals; and ${l}_a^{DF}$ is the size of the dementia-free (*DF*) population at exact age *a*. In other words, Π_*a*_ is the sum of all dementia cases occurring above age *a*, divided by the number of dementia-free persons at age *a*.

Also of interest is dementia-free life expectancy—that is, the average number of years a randomly chosen person age *a* can expect to live free of dementia—under current rates:

2 $$ {DFLE}_a=\frac{\sum_{x=a}^w{}_1L_x^{DF}}{l_a}, $$

where *DFLE* is dementia-free life expectancy; *a*, *x*, and *w* are as previously defined; ${}_1L_x^{DF}$ are person-years lived in a dementia-free (*DF*) state from age *x* to age *x* + 1; and *l*_*a*_ is the size of the entire population aged *a*, obtained from a national life table.

One can also define *conditional* dementia-free life expectancy as the average number of years that a *dementia-free person* of a given age can expect to live free of dementia:

3 $$ DFL{E}_a^{\prime }=\frac{\sum_{x=a}^w{}_1L_x^{DF}}{l_a^{DF}}, $$

where *DFLE* ^′^ indicates conditional dementia-free life expectancy; the numerator is the same as in Eq. (2); and ${l}_a^{DF}$ is the size of the *dementia-free* population aged *a.* This quantity is valuable because the number of dementia-free person-years lived above age *a* for someone who already has dementia at age *a* is 0, but the people contributing 0 dementia-free person-years to the numerator in Eq. (2) are still included in the denominator in Eq. (2).

The quantities on the right side of Eqs. (1), (2), and (3) are all multiple-decrement life table quantities, obtained using the approach in Preston et al. (2001: chapter 4).

Age-specific incidence is estimated with the following equation:

4 $$ \mathrm{logit}\left({h}_x\right)=\upalpha +\upbeta x, $$

where *h* is the incidence rate, *x* is exact age, and α and β are parameters to be estimated. The model broadly conforms to the functional form of the age patterns of Alzheimer’s disease rates (Brookmeyer and Gray 2000; Brookmeyer et al. 2011; Ziegler-Graham et al. 2008).

The model is fit using a discrete-time logistic regression on a person-year data file (Allison 1984), using the 456 subjects followed longitudinally and the longitudinal survey weight provided by ADAMS. Each subject’s longitudinal survey weight is proportional to the subject’s predicted probability of attrition, which is calculated based on observed data in HRS and ADAMS on cognitive status, general health status, and sociodemographic information (Heeringa et al. 2009; Plassman et al. 2011). Age of dementia onset was reported in completed years; thus, for incident cases, the exact age at incidence was set at the reported age (last birthday) of onset plus 0.5. Subjects who never received a diagnosis of dementia from ADAMS investigators, including those who died without a dementia diagnosis, were censored. Among the censored subjects, those whose status at the end of the ADAMS study period was *alive, dementia-free* contributed dementia-free person-years up to and including their exact age (in months) at their last assessment. A sensitivity analysis censored dementia-free survivors at the end of the ADAMS study period rather than at their last assessment.

Censored subjects whose status at the end of ADAMS was *died without dementia* contributed dementia-free person years until their exact age at death. For example, if a subject’s status at the end of ADAMS was *died without dementia*, and she died at age 78 and 5 months, then she contributed person-years of exposure until she was 78.41666. Her death would be assigned to the interval between exact ages 78.0 and 79.0. The approach of carrying the last assessment of deceased individuals forward until death is consistent with previous ADAMS reports (Plassman et al. 2011) and recommendations based on simulations of censored time-to-dementia data (Leffondré et al. 2013). It is based on the idea that if the deceased individuals had survived and developed dementia, the investigators could have been able to observe their dementia onset; decedents were therefore at risk of dementia onset until their deaths.

Considerable evidence in the literature suggests that age-specific incidence rates of dementia do not vary by sex (Chêne et al. 2015; Plassman et al. 2011; Ruitenberg et al. 2001). When a sex term was included in Eq. (4), its coefficient was statistically insignificant (*p* > .20). This insignificant result further justifies the pooling of males and females in the estimation of dementia incidence.

To estimate an age pattern of differential mortality, a Gompertz equation was fit with a Poisson regression on a person-year data file (Loomis et al. 2005), again using the longitudinal sampling weights provided by ADAMS investigators. Dementia status was modeled as a time-varying indicator to incorporate both baseline prevalent cases and incident cases (Palloni and Thomas 2013). The model is

5 $$ \ln \left({m}_{x,dem}\right)=\upalpha +{\upbeta}_1x+{\upbeta}_2dem+{\upbeta}_3x\times dem, $$

where *m* is the death rate, *x* is exact age, and *dem* is an indicator equal to 1 if the subject had dementia and equal to 0 otherwise. The parameters to be estimated from the data should be understood as follows: α represents the level of mortality in the entire population; β_1_ represents the age-pattern of mortality in the entire population; β_2_ represents the extent to which those with dementia die at a different (presumably higher) rate than those without dementia, regardless of age; and β_3_ is a parameter allowing differential mortality to vary by age.

As with the estimation of dementia incidence discussed earlier, subjects who died without a dementia diagnosis during the ADAMS study period contribute dementia-free person years until their exact age at death, and subjects who survived ADAMS without a dementia diagnosis contributed dementia-free person years until their last ADAMS assessment. A sensitivity analysis censored dementia-free survivors at the end of the ADAMS study period. Mortality data for the period after ADAMS (2009 to 2011) was used only for those with a dementia diagnosis whose state could not change until death. Not using mortality data from the post-ADAMS period for individuals without a dementia diagnosis avoids large misclassification errors whereby persons who develop dementia subsequent to ADAMS would wrongly contribute deaths without dementia and person-years without dementia to the calculations.

Based on Eq. (5), the ratio of the mortality rate among persons with dementia to that among persons without dementia—also known as the mortality rate ratio (*RR*)—is

6 $$ {RR}_x=\frac{ \exp \left(\upalpha +{\upbeta}_1x+{\upbeta}_2+{\upbeta}_3x\right)}{ \exp \left(\upalpha +{\upbeta}_1x\right)}= \exp \left({\upbeta}_2+{\upbeta}_3x\right), $$

where *x* is exact age. In this way, the *ratio* of the two mortality rates is estimated from the ADAMS sample, but the actual values of the mortality rates can be adjusted to match national data with many more deaths using national life tables.

Consistent with most of the literature, the ratio of mortality rates between those with and those without dementia were held constant across sex (Agüero-Torres et al. 1999; Garcia-Ptacek et al. 2014; Johnson et al. 2007; Lönnroos et al. 2013; Meller et al. 1999; Villarejo et al. 2011; Witthaus et al. 1999). When a sex term and an interaction term for sex by dementia status were included in Eq. (5), the coefficient on interaction term was not statistically significant (*p* > .30), providing additional justification for keeping differential mortality constant across sex. As with the modeling of incidence rates, pooling males and females to estimate differential mortality is useful with a small sample size, as in ADAMS. In this model, the only quantity that differed by sex was the overall level of age-specific mortality in the entire U.S. population.

For a given age, the mortality rate for the entire population can be decomposed into a weighted average of mortality rates of the diseased and disease-free populations, weighted by the age-specific prevalence of the disease:

7 $$ \begin{array}{l}{}_1m_x={}_1m_x^D\times {}_1P_x+{}_1{}^{death}m_x^{DF}\times \left(1-{}_1P_x\right)\\ {}={}_1{}^{death}m_x^{DF}{\times}_1{RR}_x\times {}_1P_x+{}_1{}^{death}m_x^{DF}\times \left(1-{P}_x\right),\end{array} $$

where _1_ *m*_*x*_ is the death rate in the entire population in the age interval *x* to *x* + 1; ${}_1m_x^D$ is the death rate in the same age interval for those with dementia; _1_ *P*_*x*_ is the prevalence of dementia in that age interval; ${}_1{}^{death}m_x^{DF}$ is the death rate in the age interval for the dementia-free population; and _1_ *RR*_*x*_ is the ratio of mortality rates (with-dementia vs. dementia-free) in the age interval.

The terms can be rearranged to solve for the mortality rate in the dementia-free population:

8 $$ {}_1{}^{death}m_x^{DF}=\frac{{}_1m_x}{\left({}_1P_x\times {}_1R{R}_x+1-{}_1P_x\right)} $$

and in the population with dementia:

9 $$ {}_1m_x^D={}_1{}^{death}m_x^{ND}\times {}_1R{R}_x, $$

where the overall mortality rate, _1_ *m*_*x*_, comes from the national life table; the mortality rate ratio (_1_ *RR*_*x*_) is from Eq. (6); and the age-specific prevalence is the proportion of survivors to the middle of the age interval who have dementia, as detailed in the appendix.

Using the incidence rates estimated in Eq. (4) and the mortality rates found in Eqs. (8) and (9), a multiple-decrement life table is constructed for the population without dementia, including the crucial quantity of dementia-free person-years lived in each single-year age interval, ${}_1L_x^{DF}$. The approach incorporates elements of the increment-decrement life table to keep track of a model population with dementia. Single-year age groups are used, and no recovery from dementia is allowed. The life table relations used are developed in Preston et al. (2001: chapter 4) and shown in detail in the appendix.

After the multiple-decrement life table is completed, the summary quantities of interest—risk of developing dementia, unconditional expectancies, and conditional dementia-free life expectancy—can be calculated as in Eqs. (1)–(3).

### Assessing the Effects of Long-Term Declines in Mortality

The analysis is first conducted using sex-specific life tables for the 1920 birth cohort, which was aged 81 to 88 during ADAMS, putting it in the middle of the age range of ADAMS participants. Then the same analysis was done using the 1940 birth cohort life table to show the risks of developing dementia and DFLE for a contemporary cohort—one with lower mortality rates at every age than the 1920 cohort—faced with the incidence and mortality rate ratios observed in ADAMS. The comparison of the 1920 with the 1940 cohort quantifies the effect of reductions in the overall level of mortality in the population on estimates of risk of developing dementia.

### Simulated Delays or Reductions in Dementia Incidence

A great deal of ongoing research, both privately and publicly funded, is developing treatments to delay AD and other dementias (Zissimopoulos et al. 2015), so the life cycle effects of different interventions that delay the onset of dementia are also estimated. A recent economic analysis of estimating a delayed onset of AD considered a five-year delay, which is treated here as the most optimistic of several scenarios of delayed dementia (Zissimopoulos et al. 2015). In the first scenario, the intervention delays dementia onset by one year and is effective for 50 % of the dementia-free population at age 70. In the second scenario, the same intervention affects 90 % of dementia-free 70-year-olds. In the third scenario, the intervention delays dementia onset by five years and is effective for 50 % of dementia-free 70-year-olds, and the fourth scenario delays dementia onset by five years for 90 % of dementia-free 70-year-olds. These interventions are modeled by splitting the model dementia-free population in half (or, for the second and fourth scenarios, into 10 %/90 % groups), subjecting the first group to the dementia incidence rates as modeled in Eq. (4), and subjecting the second group to the dementia incidence rates as modeled by

10 $$ \mathrm{logit}\left({h}_x^{\prime}\right)=\upalpha +\upbeta \left(x-K\right), $$

where *K* is the number of years of delay of dementia onset induced by the intervention, and the other quantities are as defined in Eq. (4). This equation assigns what had been the age 70 incidence rate to age 70 + *K*, what had been the age 71 incidence rate to age 71 + *K*, and so forth.

Another type of intervention would reduce the risk of dementia at every age rather than delaying its onset. Such an intervention generates an incidence equation such as

11 $$ \mathrm{logit}\left({h}_x^{{\prime\prime}}\right)=\upalpha +\left(\upbeta k\right)x, $$

where *k* is a value between 0 and 1 that represents the extent to which dementia incidence rises less steeply with age due to the intervention, and the other quantities are as in Eq. (4). The closer *k* is to 0, the more effective is the intervention in the sense of reducing the acceleration of dementia incidence. An intervention is simulated where *k* = 0.9 in order to reduce the (logit of) acceleration of dementia incidence with age by 10 %.

Both the dementia-free and with-dementia populations are subject to the same mortality rates as in the original analysis, based on the 1920 cohort life tables and estimated mortality rate ratios. However, the changing sizes of these two populations resulting from the simulated intervention are assumed to change the overall mortality rate (Eq. (7). These simulations illustrate the effects of possible future reductions in dementia incidence; they are not projected outcomes based on expected rates.

### Estimation of Standard Errors and Confidence Intervals

To generate standard errors and confidence intervals around the lifetime probability and expectancy estimates, the parameter estimates generating the age-specific dementia incidence schedules (the fitted values of [α β] in Eq. (4)) and differential mortality (the fitted values for [α β_1_ β_2_ β_3_] in Eq. (5)) are considered stochastic. Total mortality, derived from the SSA cohort life tables, was treated as deterministic (i.e., having 0 variance) (Abatih et al. 2008; Loukine et al. 2012); and the life table assumptions, such as linearity of survival within age intervals, were also considered not to contribute any additional variance.

For dementia incidence, the estimates of [α β] in Eq. (4), along with their associated variance-covariance matrix, were used as the parameters of a bivariate normal distribution to draw 1,000 independent values of [α β], generating 1,000 incidence schedules. Separately, an analogous procedure with the estimated parameters and variance-covariance matrix from Eq. (4) or Eq. (5) was used to generate 1,000 age schedules of the mortality rate ratio between those with and those without dementia. Each incidence schedule was paired with one schedule of the mortality rate ratios and run through the life table operations, producing 1,000 dementia probability and expectancy estimates. Tables show the means and standard errors (square roots of variances) of the 1,000 estimates (Fishman 2015; Mooney 1997; Salomon et al. 2001).

Parameters from Eqs. (4) and (5) were estimated using Stata version 14 (StataCorp 2015), using first-order Taylor Series linearization for variance estimation with the *svy* routine with longitudinal sampling weights provided by ADAMS (Heeringa et al. 2009). Random sampling for the estimation of standard errors was conducted in R using the *mvrnorm* command in the MASS package (Venables and Ripley 2002), and life table operations were conducted using base R (R Core Team 2014). The HRS and ADAMS data are available to the public after a registration procedure (HRS 2013).

## Results

There were 308 cases of dementia at baseline out of 856 unweighted sample members. All baseline sample members were at risk of death, generating 519 deaths in 3,520 person-years at risk. Among the 456 individuals without dementia at baseline who were followed longitudinally for dementia onset, 106 developed dementia in 2,142 person-years at risk. Of the 106 incident cases, 29 were *interval-censored*, meaning they were assigned an age at onset between adjacent ADAMS examinations rather than their age at a particular examination as described in the section, Sample and Definitions. The estimates of the regression parameters in Eqs. (4) and (5), along with the variance-covariance matrices used to sample the 1,000 simulated incidence and mortality rate–ratio schedules for the confidence intervals, are shown in Table 1.

**Table 1:** Parameter estimates in model of dementia incidence (*h*) and in the model of mortality (*m*) by dementia status and age

Term	Coefficient Estimate	Variance-Covariance Matrix			
		Age			Constant
A. Model of Dementia Incidence					
Age (*x*)	0.087151	0.000407			
Constant term	–10.6868	–0.03363			2.793747
Term		Variance-Covariance Matrix			
		Dementia	Age	Age × *Dem*	Constant
B. Model of Mortality by Dementia Status and Age					
Dementia (*Dem*)	6.435545	3.427942			
Age (*x*)	0.110955	0.027964	0.000299		
Age × *Dem*	–0.06139	–0.03975	–0.00033	0.000464	
Constant term	–12.2631	–2.35307	–0.025	0.027693	2.10715

*Notes:* See the text for details of model fitting. Model of dementia incidence: logit(*h*_*x*_) = α^′^ + β^′^ *x*.Model of mortality by dementia status and age: ln(*m*_*x* , *dem*_) = α + β_1_ *x* + β_2_ *dem* + β_3_ *x* × *dem*. Longitudinal sampling weight ADLONGWT was used in model fitting. Data are from the Aging, Demographics, and Memory Study (ADAMS), United States, 2001–2009. For prevalence and differential mortality, *n* = 856. For incidence, *n* = 456. Data on mortality by dementia status are from Health and Retirement Study (HRS), United States, 2001–2011.

Table 2 shows the fitted age-specific dementia incidence rates, with 16 new cases per 1,000 person-years at age 75; 37 new cases per 1,000 person-years at age 85; and 56 to 86 new cases per 1,000 person-years at ages 90–95. The incidence rates shown here are close to those previously reported from ADAMS (Plassman et al. 2011) albeit not exactly the same because of this study’s use of the fitted values for each single-year age rather than the observed values for large age groups. The age pattern of mortality rate ratios shown in Table 2, showing a rapid decline in differential mortality with age, is largely consistent with that found in other, nonnational and non-U.S. samples, although pace of decline of differential mortality with age varies widely in the literature (Guehne et al. 2005; Ostbye et al. 1999; Tschanz et al. 2004; Villarejo et al. 2011).

**Table 2:** Estimated dementia incidence and mortality rate ratios (RR)

Age	Incidence	SE	RR^a^	SE^b^
70	0.010	0.003	8.86	3.344
75	0.016	0.003	6.37	1.761
80	0.024	0.003	4.63	0.896
85	0.037	0.005	3.41	0.513
90	0.056	0.011	2.54	0.447
95	0.086	0.023	1.91	0.479
100	0.130	0.042	1.48	0.472

*Notes:* Data are from the Aging, Demographics, and Memory Study (ADAMS), United States, 2001–2009. For mortality rate ratios (RR), *n* = 856. For incidence, *n* = 456. Mortality data are from the U.S. Social Security Administration life tables for 1920 birth cohort. Mortality by dementia status data are from the Health and Retirement Study (HRS), United States, 2001–2011. Parametric models were fitted to incidence and mortality data from ADAMS to generate single-year age-specific estimates.

^a^ RR = Ratio of rates of death, with dementia versus without dementia.

^b^ SE = Standard error.

The sensitivity analysis censoring dementia-free subjects at the end of the ADAMS study period (shown in Online Resource 1) produced incidence rates that were considerably lower than those previously reported in ADAMS. It also produced mortality rate ratios considerably higher than found elsewhere in the literature (Beeri and Goldbourt 2011; Ostbye et al. 1999; Tschanz et al. 2004; Villarejo et al. 2011). These results were expected because the sensitivity analysis added person-years lived free of dementia without adding new dementia cases or new person-years lived with dementia. Despite their discordance with the literature, the results of the sensitivity analysis show that the main results potentially underestimate person-years lived free of dementia.

Table 3 shows the estimates of the life table quantities of interest for the 1920 birth cohort. The average 70-year-old male has a 26.9 % probability (SE = 3.2 %) of developing dementia in his remaining lifetime and will live an expected 0.76 years (SE = 0.22 years) with dementia. For an average 70-year-old female, the risk of developing dementia in her remaining life is 34.7 % (SE = 3.7 %), and she will live an expected 1.74 years (SE: 0.29 years) with dementia. For males, the risk of developing dementia and life expectancy with dementia barely decline with age; at age 95, dementia-free males have an estimated 24.4 % probability of developing dementia before death and an expected 0.76 years lived with dementia. The absence of an age trend in the probability of developing dementia for males reflects a dementia incidence rate that rises with age but is essentially offset by the rising mortality rate. Females experience some decline with age in the estimated probability of developing dementia and in life expectancy with dementia (DLE), to 28.1 % and 1.12 years, respectively, at age 95. Again, it is important to note that these life expectancies with dementia pertain to the entire population, not just those with dementia. Meanwhile, dementia-free life expectancy, both unconditional and conditional, fall sharply with age for both sexes, in step with total life expectancy.

**Table 3:** Estimated life table quantities

Age	LE	DFLE	DLE	SE	Probability of Dementia	SE	DFLE′	SE
A. Males								
70	12.31	11.55	0.76	0.220	0.269	0.032	11.91	0.190
75	9.65	8.90	0.75	0.228	0.271	0.032	9.35	0.154
80	7.26	6.51	0.76	0.223	0.267	0.036	7.04	0.123
85	5.20	4.47	0.73	0.211	0.257	0.044	5.04	0.094
90	3.64	2.93	0.72	0.200	0.247	0.056	3.49	0.080
95	2.61	1.85	0.76	0.200	0.244	0.072	2.43	0.082
100	2.02	1.10	0.92	0.207	0.255	0.091	1.77	0.084
B. Females								
70	15.25	13.51	1.74	0.292	0.347	0.037	13.93	0.278
75	11.91	10.21	1.69	0.303	0.341	0.038	10.88	0.213
80	8.91	7.34	1.56	0.300	0.329	0.043	8.15	0.172
85	6.37	4.99	1.38	0.285	0.312	0.052	5.84	0.133
90	4.42	3.21	1.21	0.262	0.293	0.064	4.02	0.101
95	3.10	1.97	1.12	0.243	0.281	0.079	2.76	0.088
100	2.32	1.13	1.19	0.228	0.284	0.097	1.97	0.086

*Notes:* LE = total life expectancy for a randomly chosen person in the population of given age. DFLE = dementia-free life expectancy for a randomly chosen person in the population of given age. DLE = life expectancy with dementia for a randomly chosen person in the population of given age. Probability of dementia = probability that a dementia-free person will develop dementia later in life. DFLE′ = dementia-free life expectancy for a dementia-free person of given age. By construction, DFLE and DLE have the same standard error, and LE has zero variance. Quantities are calculated using the fitted values of dementia incidence and relative risk of death (with dementia vs. without) shown in Table 2. Data are from the Aging, Demographics, and Memory Study (ADAMS), United States, 2001–2009. For relative risk of death, *n* = 856. For incidence, *n* = 456. Mortality data are from the U.S. Social Security Administration life tables for the 1920 birth cohort. Data on mortality by dementia status are from the Health and Retirement Study (HRS), United States, 2001–2011.

### Effect of Declining Mortality

Using the 1940 cohort life table rather than that of 1920 raises risk estimates at all ages (Table 4). The estimated increase at age 70 is 3.9 percentage points for males and 2.7 percentage points for females. The probability that a dementia-free 70-year-old male from this cohort develops dementia later in life is approximately 30.8 % (SE = 3.4 %); for a dementia-free 70-year-old female, it is 37.4 % (SE = 3.8 %). The increase in dementia risk results from population-wide reductions in mortality between the two birth cohorts, reducing the competing risk of death and allowing a larger proportion of the population to survive to ages of high dementia incidence. As with the 1920 cohort table, life expectancy with dementia barely declines with age for males, from 1.10 years at age 70 to 0.97 years at age 95; it declines slightly for females, from 1.97 years at age 70 to 1.40 years at age 95. The 1940 cohort results illustrate that individuals in younger, lower-mortality cohorts face higher age-specific remaining lifetime risks of dementia than individuals in older, higher-mortality cohorts. The percentage increase in risk across the two cohorts is larger for males than females because females have lower mortality than males to begin with (a larger base leads to smaller percentage change), and/or because mortality declined less for females than for males between these two cohorts (Preston and Wang 2006).

**Table 4:** Estimated life table quantities using 1940 cohort life table

Age	LE	DFLE	DLE	SE	Probability of Dementia	SE	DFLE′	SE
A. Males								
70	13.64	12.54	1.10	0.262	0.308	0.034	12.93	0.237
75	10.65	9.57	1.08	0.272	0.306	0.035	10.13	0.192
80	7.96	6.93	1.03	0.268	0.298	0.040	7.59	0.156
85	5.70	4.74	0.96	0.253	0.286	0.049	5.44	0.121
90	4.05	3.13	0.93	0.239	0.276	0.063	3.82	0.101
95	2.95	1.97	0.97	0.233	0.273	0.080	2.68	0.098
100	2.30	1.15	1.14	0.227	0.285	0.099	1.96	0.095
B. Females								
70	15.99	14.01	1.97	0.332	0.374	0.038	14.44	0.315
75	12.62	10.67	1.95	0.343	0.370	0.041	11.38	0.252
80	9.57	7.73	1.84	0.340	0.359	0.047	8.62	0.209
85	6.93	5.26	1.66	0.324	0.342	0.057	6.23	0.166
90	4.89	3.40	1.49	0.301	0.325	0.070	4.35	0.129
95	3.49	2.09	1.40	0.277	0.313	0.087	3.02	0.109
100	2.63	1.18	1.46	0.248	0.316	0.105	2.16	0.101

*Notes:* LE = total life expectancy for a randomly chosen person in the population of given age. DFLE = dementia-free life expectancy for a randomly chosen person in the population of given age. DLE = life expectancy with dementia for a randomly chosen person in the population of given age. Probability of dementia = probability that a dementia-free person will develop dementia later in life. DFLE′ = dementia-free life expectancy for a dementia-free person of given age. By construction, DFLE and DLE have the same standard error, and LE has zero variance. Quantities are calculated using fitted values of dementia incidence and relative risk of death (with dementia vs. without) shown in Table 2. Mortality rates for total population come from the U.S. Social Security Administration 1940 birth cohort life tables. Data come from the Aging, Demographics, and Memory Study (ADAMS), United States, 2001–2009. For relative risk of death, *n* = 856. For incidence, *n* = 456. Mortality data come from the U.S. Social Security Administration life tables for the 1940 birth cohort. Data for mortality by dementia status come from the Health and Retirement Study (HRS), United States, 2001–2011.

### Effect of Delaying Onset of Dementia

Table 5 shows the estimates for dementia risk associated with the scenarios in which dementia incidence is delayed or reduced. (The age patterns of incidence in each scenario are presented in Online Resource 1.) Table 5 should be compared with the Probability of Dementia column and its standard error in Table 3, isolating the effect of declines or delays in dementia incidence on the life table quantities. In the first two scenarios, an intervention delays the risk of dementia by one year; in Scenario 1, the intervention affects 50 % of dementia-free 70-year-olds, and in Scenario 2, it affects 90 %. The estimates for Scenarios 1 and 2 in Table 5 indicate that this intervention would reduce remaining lifetime risk at age 70 by only 1 to 2 percentage points, with similar reductions at older ages. The small difference between Scenarios 1 and 2 shows that the proportion of the population aged 70 for which this intervention is effective has a small effect on the estimates. Extending the reach from 50 % to 90 % of dementia-free 70-year-olds reduces dementia risk by less than 1 percentage point.

**Table 5:** Estimated remaining lifetime risk of dementia under intervention scenarios

	Scenario 1^a^		Scenario 2^b^		Scenario 3^c^		Scenario 4 ^d^		Scenario 5^e^	
Age	Estimate	SE	Estimate	SE	Estimate	SE	Estimate	SE	Estimate	SE
A. Males										
70	0.261	0.032	0.255	0.032	0.232	0.032	0.202	0.032	0.213	0.031
75	0.263	0.031	0.256	0.031	0.233	0.029	0.203	0.028	0.212	0.028
80	0.259	0.034	0.253	0.033	0.229	0.030	0.199	0.026	0.205	0.026
85	0.249	0.042	0.243	0.041	0.219	0.035	0.192	0.029	0.194	0.029
90	0.240	0.054	0.234	0.052	0.209	0.044	0.184	0.038	0.181	0.035
95	0.236	0.069	0.231	0.068	0.205	0.057	0.182	0.051	0.171	0.043
100	0.247	0.088	0.242	0.087	0.213	0.074	0.193	0.068	0.170	0.052
B. Females										
70	0.338	0.036	0.330	0.036	0.302	0.035	0.266	0.034	0.276	0.034
75	0.331	0.037	0.324	0.036	0.296	0.034	0.260	0.032	0.268	0.031
80	0.320	0.042	0.312	0.041	0.284	0.036	0.250	0.032	0.255	0.031
85	0.303	0.050	0.296	0.049	0.268	0.042	0.236	0.036	0.237	0.035
90	0.285	0.062	0.278	0.060	0.251	0.052	0.222	0.045	0.217	0.041
95	0.273	0.077	0.267	0.075	0.239	0.064	0.214	0.058	0.200	0.049
100	0.277	0.094	0.271	0.093	0.240	0.080	0.218	0.074	0.192	0.057

*Notes:* Remaining-lifetime risk is the probability that a dementia-free person of a given age will develop dementia later in life. Original estimates of incidence are shown in Table 2; incidence under interventions are shown in Online Resource 1. Mortality data come from the U.S. Social Security Administration life tables for the 1920 birth cohort.

^a^ Scenario 1: Dementia incidence delayed by one year, effective for 50 % of dementia-free population age 70.

^b^ Scenario 2: Dementia incidence delayed by one year, effective for 90 % of dementia-free population age 70.

^c^ Scenario 3: Dementia incidence delayed by five years, effective for 50 % of dementia-free population age 70.

^d^ Scenario 4: Dementia incidence delayed by five years, effective for 90 % of dementia-free population age 70.

^e^ Scenario 5: Acceleration of dementia incidence with age reduced by 10 %.

A larger reduction in dementia risk is achieved by an intervention that delays dementia onset by five years and reaches 50 % of the dementia-free population at age 70 (Scenario 3); now the reduction is 3.7 percentage points for males and 4.5 for females. If this five-year delay affected 90 % of dementia-free 70-year-olds (Scenario 4), it would reduce dementia risk at age 70 by 6.7 percentage points for males and 8.1 percentage points for females—a 25 % reduction in remaining lifetime risk for males and a 23 % reduction for females. Similar reductions in remaining lifetime risk are achieved by an intervention that reduces the rate of acceleration of dementia incidence with age, as in Scenario 5. This intervention achieves a 5.6 percentage point reduction in risk for males and 7.1 percentage point reduction for females. Reducing the rate of acceleration of dementia incidence with age is also the only intervention that produces a markedly downward-sloping age pattern of dementia risk for males.

## Discussion

This study provides the first nationally representative estimates of the probability that an average dementia-free person at various ages will develop dementia before death. These estimates suggest that approximately 27 % of dementia-free 70-year-old males and approximately 35 % of dementia-free 70-year-old females in the 1920 birth cohort will develop dementia before they die. For the 1940 birth cohort, these estimates rise to about 31 % for males and 37 % for females. The expected number of years that a randomly chosen individual aged 70 could expect to live with dementia is approximately 0.75 years for males and 1.75 years for females in the 1920 birth cohort, rising to 1.1 years for males and 2.0 years for females in the 1940 cohort. These estimates imply a larger need for individuals and families to plan for a life stage with dementia than that implied by previous community-based studies of dementia risk.

The median household headed by someone aged 55–64 in 2010 (i.e., people born in the 1940s and early 1950s) had just $12,000 in retirement savings according to the U.S. Federal Reserve’s National Survey of Consumer Finances (Rhee 2013). Such a low figure suggests that the typical U.S. household does not have nearly enough savings to pay for the care of someone with dementia, even for a year or two. This savings figure does not include long-term care insurance policies, but sales of such policies have declined in recent years, and these policies no longer offer true catastrophic coverage (Favreault et al. 2015). These facts suggests that Medicaid would, under current law, likely be expected to cover the costs of long-term care for many of these elderly persons. A recent report from the Centers for Medicare and Medicaid Services (CMS) projected increases in costs per enrollee due to population aging, but it did not project an increase in the growth rate of Medicaid enrollment, nor did it project an increase in the growth rate of Medicaid spending specifically on nursing care facilities and continuing care retirement communities (Keehan et al. 2016). The low household savings among 1940s and 1950s birth cohorts, their large initial size (i.e., early Baby Boomers), and the increases in dementia risk associated with reduced mortality, as shown in this study, combine to suggest possible future spikes in Medicaid enrollment among the elderly.

Although Medicaid could play an increasing role in long-term care for those with dementia, family members provide the majority of care needed by persons with dementia (Adelman et al. 2014; Hurd et al. 2013). People who care for family members with dementia face greater health risks than age-contemporaries who care for relatives without dementia, and these health risks seem to persist even after the care recipients have died (Dassel and Carr 2016; Fisher et al. 2011). One important implication of the present study’s results is that elderly persons do not “age out” of the risk of developing dementia because increases in dementia incidence with age roughly keep pace with increases in mortality rates. This finding is perhaps most clearly illustrated in the lack of decline with age in life expectancy with dementia (DLE), especially for males. Yet, as individuals age, they are decreasingly likely to have surviving spouses who are able to care for them. The lack of a decline in DLE with age and the increase in DLE across successively lower-mortality birth cohorts thus imply that adult children, especially daughters (Fisher et al. 2011; Kasper et al. 2015), increasingly shoulder dementia caregiving responsibilities for long durations, with potential negative consequences for their mental health (Fisher et al. 2011) and labor force participation (Hurd et al. 2013).

On a more positive note, although dementia-free life expectancy declines rapidly with age, dementia-free individuals of both sexes and at all ages are expected to live the majority of their remaining lives free of dementia. Furthermore, the simulations (particularly Scenarios 3 and 4, simulating a five-year delay) demonstrate that interventions that “merely” delay dementia onset can greatly lower the percentage of people who will ever develop dementia because the competing risk of death is high at these ages. Interventions that slow the acceleration of dementia incidence with age, and thus make remaining lifetime risk of dementia decline with age (as in Scenario 5), also have increasing potential to reduce the fraction of people who will ever develop dementia because the total population consists of an increasing share of very elderly people.

A recent study, Adult Changes in Thought (ACT) (Tom et al. 2015), reported dementia-free life expectancy for dementia-free cohort members (DFLE′) age 70, estimating 14.3 years for males and 15.7 years for females. The present study’s estimates were 11.1 years for males and 13.4 years for females (Table 3). Because the ACT cohort had much longer life expectancy overall—for example, 16.0 years for males age 70 versus 12.3 years in the national population—it is not surprising that it also had longer conditional dementia-free life expectancy. More broadly, the fact that the ACT cohort’s life expectancy was so much higher than that of the U.S. cohorts under study here suggests that the ACT cohort was in substantially better health than a nationally representative cohort of their age contemporaries would be. This difference in health status likely explains the discrepancies between the ACT study and this ADAMS-based study. The ACT study did not specifically report on lifetime probability of developing dementia.

Another past study of an individual’s remaining lifetime risk of dementia that incorporated a competing-risks framework used Framingham data from 1975–1995 (Seshadri et al. 1997). It estimated that a dementia-free male aged 65 had a 14.3 % probability of developing dementia at some point in his remaining life, and a dementia-free female aged 65 had a 21.7 % probability of developing dementia at some point in her remaining life (Seshadri and Wolf 2007). One reason why the estimates of the probability of developing dementia presented here are considerably higher than the Framingham-based estimates is that overall mortality during ADAMS was lower than overall mortality during Framingham. The age-standardized mortality rate (ASMR) in the U.S. population aged 65 and older in 2005—the middle of the ADAMS study period—was 4,804 deaths per 100,000 person-years lived. This rate was much lower than the ASMR in Massachusetts for ages 65 and older in 1985—the middle of Framingham’s study period—which was 5,679 deaths per 100,000 person-years lived (Centers for Disease Control and Prevention 2014). The comparison of the 1920 SSA cohort table to that of 1940 demonstrated that lower mortality levels imply higher remaining lifetime risks of dementia.

The estimates of dementia risk presented here fall between those found in Framingham and those estimated for a national sample from Canada, where the authors estimated that slightly more than 40 % of 70-year-olds in Canada would develop dementia before death (Carone et al. 2014). The dementia incidence rates found in the Canadian study were higher than those estimated here (Canadian Study of Health and AgingWorking Group 2000), producing higher estimates of dementia risk. Incidence of dementia as measured in Canada could be higher than that measured in ADAMS because of actual differences in dementia incidence between the Canadian and U.S. elderly populations, or because of differences between the two studies in the method of ascertaining dementia status. For example, the Canadian study diagnosed new cases of dementia posthumously for some subjects, based on family reports of cognitive status three months before death (Canadian Study of Health and AgingWorking Group 2000). No posthumous assessments were made in ADAMS.

A recent study using HRS produced somewhat higher estimates of unconditional life expectancy with dementia than those reported here (Crimmins et al. 2016). Two factors explain the differences. First, Crimmins et al. used the 2000 and 2010 period life tables, which had a higher total life expectancy at old ages than the 1920 or 1940 cohort life tables used in the present analysis. Second, using the full HRS appears to classify a larger fraction of adults of a given age as having dementia compared with the baseline ADAMS. For example, Crimmins et al. estimated that in 2000, 6.63 % of males aged 70–74 and 10.65 % of males aged 75–79 had dementia. In contrast, baseline ADAMS estimated that in October, 2002—the midpoint of the baseline ADAMS assessment period—5.25 % of males aged 71–79 had dementia (Plassman et al. 2007). Higher total life expectancy and higher dementia prevalence combined to produce higher estimates of DLE in the Crimmins et al. study than in the present study.

One limitation of the present study is that it misses cases of dementia that develop prior to age 70. However, the incidence of such early-onset dementia is rare; studies from other countries estimate it is on the order of one to two cases per 1,000 person-years lived (Schrijvers et al. 2012). An additional limitation is that although the ADAMS staff were able to ascertain an age at onset during interwave periods, there is potential for measurement error for subjects assigned an age of onset that falls between their ages at consecutive survey waves, especially for subjects not assessed in Wave B. Furthermore, the small sample size prevented the estimation of reasonably precise parameter estimates for subgroups, such as African Americans or those who did not complete high school. The purpose of using the small ADAMS sample, rather than the larger HRS sample, was to use the clinically validated diagnoses of dementia available only in ADAMS. The differences in dementia prevalence estimates between the recent HRS-based study and the present study, detailed in the previous paragraph, underscore the value of using the clinical diagnoses.

A final limitation relates to the higher response rates and slower attrition of healthy subjects. If the sampling weights do not fully correct for differential selection and attrition by health and cognitive status, then the number of dementia-free person-years lived in the population could be overestimated and the number of new dementia cases could be underestimated, thus underestimating incidence rates of dementia. At the same time, if sample members with dementia have lower mortality (relative to those without dementia) than nonsample members with dementia because of a more favorable distribution of comorbidities, then the mortality rate ratios in this study could also be underestimated. The overall effect of differential attrition that is not corrected by sampling weights would thus be to underestimate the remaining lifetime risk of developing dementia.

The results shown here suggest that a large fraction of current and near-future elderly—and a fraction perhaps 50 % higher than that implied by the Framingham cohorts—will develop dementia in their remaining lifetimes. Furthermore, as old-age mortality declines, remaining lifetime risks of dementia increase substantially across birth cohorts. The results further show that if treatments delaying or reducing dementia risk become widespread, this fraction can be reduced by perhaps 7 percentage points, or approximately 25 %, in an optimistic scenario. Given the high costs per person with dementia, a 25 % reduction in remaining lifetime risk of dementia amounts to a large economic gain. Time trends observed in Framingham do provide a reason for optimism because the most recent evidence has shown that dementia incidence declined from the 1970s to the 2000s in the Framingham cohorts (Satizabal et al. 2016)—a decline that might have occurred nationally as well (Crimmins et al. 2016; Langa et al. 2017).

### Electronic supplementary material

Online Resource 1 (PDF 665 kb)

## Appendix: Multiple-Decrement Life Table Relations

The overall rate of decrement from the dementia-free population is the dementia incidence rate, which comes from Eq. (4), plus the mortality rate for the dementia-free population:

12 $$ {}_1m_x^{DF}={}_1{}^{death}m_x^{DF}+{}_1h_x, $$

and the probability of exiting the dementia-free population at a given age, assuming decrements occur on average halfway through each age interval, is

13 $$ {}_1q_x^{DF}=\frac{{}_1m_x^{DF}}{\left(1+0.5{}_1m_x^{DF}\right)}. $$

The probability of exiting from each respective cause is

14 $$ {}_1{}^{dem}q_x^{DF}={}_1q_x^{DF}\times \left(\frac{{}_1h_x}{{}_1m_x^{DF}}\right) $$

15 $$ {}_1{}^{Death}q_x^{DF}={}_1q_x^{DF}\times \left(\frac{{}_1{}^{death}m_x^{DF}}{{}_1m_x^{DF}}\right). $$

Define ${l}_x^{DF}$ as the number of dementia-free survivors to the *x*th birthday, so that the number of exits from the dementia-free population, by type of exit, is

16 $$ {}_1{}^id_x^{DF}={l}_x^{DF}\times {}_1{}^id_x^{DF}={l}_x^{DF},\kern1.25em i=dem, death. $$

The number of dementia-free survivors to the next age is

17 $$ {l}_{x+1}^{DF}={l}_x^{DF}-{}_1{}^{dem}d_x^{DF}-{}_1{}^{death}m_x^{DF}. $$

For an approximation of the prevalence of dementia at age 70, the fitted value of prevalence for age 70.0 is obtained from baseline ADAMS (*n* = 856 subjects) using the following model:

18 $$ \mathrm{logit}\left({P}_x\right)=\upalpha +\upbeta x, $$

where *P* is prevalence, and *x* is exact age. The starting sizes (radices) of the dementia-free and with-dementia populations are

19 $$ {l}_{70}^{DF}=\left(1-{P}_{70}\right)\times {l}_{70}, $$

20 $$ {l}_{70}^D=\left({P}_{70}\right)\times {l}_{70}, $$

where the superscript *DF* indicates being in the dementia-free state, and the superscript *D* indicates being in the with-dementia state.

After age 70, the population with dementia is tracked as follows. The only way to exit the population with dementia is death, so the probability of death with dementia is

21 $$ {}_1q_x^D={}_1{}^{death}m_x^D=\frac{{}_1m_x^D}{\left(1+0.5{}_1m_x^D\right)}. $$

The size of the population reaching the *x*th birthday with dementia is defined as ${l}_x^D$, so the number of deaths is

22 $$ {}_1{}^{Death}d_x^D={l}_x^D\times {}_1{}^{Death}q_x^D. $$

However, those who develop dementia while age *x* are subject to the risk of death ${m}_x^D$ after they develop dementia. If they develop dementia halfway through the age interval, on average, then the probability of death with dementia for these new cases in that interval is

23 $$ {q_{new}^{Death}}_x^D=\frac{{}_1m_x^D}{\left(2+0.5{}_1m_x^D\right)}, $$

and the number of deaths among new dementia cases is

24 $$ {}_{new}{}^{Death}d_x^D={}_1{}^{Dem}q_x^{DF}\times {}_{new}{}^{Death}q_x^D. $$

The size of the population with dementia at the subsequent (exact) age is

25 $$ {l}_{x+1}^D={l}_x^D+{}_1{}^{Dem}d_x^{DF}-{}_1{}^{Death}d_x^D-{}_{new}{}^{Death}d_x^D. $$

Person-years lived in the dementia-free state are calculated assuming exits occur linearly within age intervals:

26 $$ {}_1L_x^{DF}={l}_{x+1}^{DF}+0.5\left({l}_x^{DF}-{l}_{x+1}^{DF}\right). $$

Person-years lived in a state of dementia are simply

27 $$ {}_1L_x^D={}_1L_x-{}_1L_x^{DF}. $$

Filling in the table for the subsequent age (*x* + 1) requires an approximation of the proportion of survivors with dementia in the middle of the age (*x* + 1, *x* + 2) interval because the mortality rates in Eqs. (7)–(9) pertain to age intervals rather than exact ages. This approximation again uses the assumption of linearity of survivorship in small intervals: it is assumed that one-half the attrition recorded from exact ages *x* to *x* + 1 will occur from exact age *x* + 1 to the middle of the (*x* + 1, *x* + 2) interval. The approximated number of persons in state *i* in the middle of the age (*x* + 1, *x* + 2) interval is denoted as ${}_1\widehat{L}_{x+1}^i$, while the *L* column for the entire population (from SSA cohort life tables) is assumed to record all survivors in the middle of the given age interval:

28 $$ \begin{array}{l}{}_1\widehat{L}_{x+1}^D={}_1L_{x+1}-{}_1\widehat{L}_{x+1}^{DF}={}_1L_{x+1}-\left[{l}_{x+1}^{DF}-0.5\left({l}_x^{DF}-{l}_{x+1}^{DF}\right)\right]\\ {}={}_1L_{x+1}-\left[1.5{l}_{x+1}^{DF}-0.5{l}_x^{DF}\right].\end{array} $$

Prevalence of dementia at the subsequent age is estimated as the proportion of mid-interval survivors living in a state of dementia:

29 $$ {}_1P_{x+1}=\frac{{}_1\widehat{L}_{x+1}^D}{{}_1L_{x+1}}, $$

which is used to solve for the mortality rate in the dementia-free population for the age *x* + 1 interval, using Eqs. (7) and (8).

Because narrow (one-year) age intervals are used, the resulting ${\widehat{L}}_x^i$ columns from Eq. (28) will be close to the ${L}_x^i$ columns from Eqs. (26) and (27). The similarity of the two columns is shown in Online Resource 1.

## References

[CR1] Abatih E, Van Oyen H, Bossuyt N, Bruckers L (2008). Variance estimation methods for health expectancy by relative socio-economic status. European Journal of Epidemiology.

[CR2] Adelman, R. D., Tmanova, L. L., Delgado, D., Dion, S., & Lachs, M. S. (2014). Caregiver burden: A clinical review. *JAMA, 311,* 1052–1060. 10.1001/jama.2014.304 24618967

[CR3] Agüero-Torres H, Fratiglioni L, Guo Z, Viitanen M, Winblad B (1999). Mortality from dementia in advanced age: A 5-year follow-up study of incident dementia cases. Journal of Clinical Epidemiology.

[CR4] Allison PD (1984). Event history analysis.

[CR5] Alzheimer’s Association (A.D.). (2014). 2014 Alzheimer’s disease facts and figures. *Alzheimer’s & Dementia, 10*(2), e47–e92. doi:10.1016/j.jalz.2014.02.001 10.1016/j.jalz.2014.02.001 24818261

[CR6] American Psychiatric Association (APA) (2000). *Diagnostic and statistical manual of mental disorders* (4th ed., Text Revision).

[CR7] Beeri MS, Goldbourt U (2011). Late-life dementia predicts mortality beyond established midlife risk factors. American Journal of Geriatric Psychiatry.

[CR8] Bell FC, Miller ML (2005). *Life tables for the United States social security area 1900–2100* (Actuarial Study No. 120).

[CR9] Brookmeyer, R., Evans, D. A., Hebert, L., Langa, K. M., Heeringa, S. G., Plassman, B. L., & Kukull, W. A. (2011). National estimates of the prevalence of Alzheimer’s disease in the United States. *Alzheimer’s & Dementia, 7,* 61–73. 10.1016/j.jalz.2010.11.007 PMC3052294 21255744

[CR10] Brookmeyer, R., & Gray, S. (2000). Methods for projecting the incidence and prevalence of chronic diseases in ageing populations: Application to Alzheimer’s disease. *Statistics in Medicine, 19,* 1481–1493. 10.1002/(sici)1097-0258(20000615/30)19:11/12<1481::aid-sim440>3.0.co;2-u 10844713

[CR11] Canadian Study of Health and Aging Working Group (2000). The incidence of dementia in Canada. Neurology.

[CR12] Carone M, Asgharian M, Jewell NP (2014). Estimating the lifetime risk of dementia in the Canadian elderly population using cross-sectional cohort survival data. Journal of the American Statistical Association.

[CR13] Centers for Disease Control and Prevention (CDC). (2014). *Compressed mortality files, 1979–1998 and 1999–2013 *[Data set]. Hyattsville, MD: National Center for Health Statistics, CDC WONDER. Retrieved from http://wonder.cdc.gov/cmf-icd10.html

[CR14] Chêne G, Beiser A, Au R, Preis SR, Wolf PA, Dufouil C, Seshadri S (2015). Gender and incidence of dementia in the Framingham Heart Study from mid-adult life. Alzheimer’s & Dementia.

[CR15] Crimmins EM, Beltrán-Sánchez H (2010). Mortality and morbidity trends: Is there compression of morbidity?. Journals of Gerontology, Series B: Psychological Sciences and Social Sciences.

[CR16] Crimmins EM, Hayward MD, Hagedorn A, Saito Y, Brouard N (2009). Change in disability-free life expectancy for Americans 70 years old and older. Demography.

[CR17] Crimmins EM, Saito Y, Kim JK (2016). Change in cognitively healthy and cognitively impaired life expectancy in the United States: 2000–2010. SSM - Population Health.

[CR18] Dassel KB, Carr DC (2016). Does dementia caregiving accelerate frailty? Findings from the Health and Retirement Study. Gerontologist.

[CR19] Favreault MM, Gleckman H, Johnson RW (2015). Financing long-term services and supports: Options reflect trade-offs for older Americans and federal spending. Health Affairs.

[CR20] Fisher, G. G., Franks, M. M., Plassman, B. L., Brown, S. L., Potter, G. G., Llewellyn, D., . . . Langa, K. M. (2011). Caring for individuals with dementia and cognitive impairment, not dementia: Findings from the aging, demographics, and memory study. *Journal of the American Geriatrics Society, 59,* 488–494. 10.1111/j.1532-5415.2010.03304.x PMC3646395 21391939

[CR21] Fishman EI (2015). *Variance estimation for a complex life table quantity: Disease-free life expectancy* (PSC Working Paper Series 15-2).

[CR22] Garcia-Ptacek S, Farahmand B, Kåreholt I, Religa D, Cuadrado ML, Eriksdotter M (2014). Mortality risk after dementia diagnosis by dementia type and underlying factors: A cohort of 15,209 patients based on the Swedish Dementia Registry. Journal of Alzheimer’s Disease.

[CR23] Guehne U, Riedel-Heller S, Angermeyer MC (2005). Mortality in dementia. Neuroepidemiology.

[CR24] He W, Larsen LJ (2014). *Older Americans with a disability: 2008−2012* (American Community Survey Reports, ACS-29).

[CR25] Health and Retirement Study (HRS) (2013). Public use dataset.

[CR26] Heeringa, S. G., Hurd, M., Langa, K. M., Ofstedal, M. B., Plassman, B. L., Rodgers, W. L., & Weir, D. R. (2009). *Sample design, weighting and analysis for ADAMS* (Report). Retrieved from http://hrsonline.isr.umich.edu/sitedocs/userg/ADAMSSampleWeights_Jun2009.pdf

[CR27] Hurd MD, Martorell P, Delavande A, Mullen KJ, Langa KM (2013). Monetary costs of dementia in the United States. New England Journal of Medicine.

[CR28] Johnson, E., Brookmeyer, R., & Ziegler-Graham, K. (2007). Modeling the effect of Alzheimer’s disease on mortality. *International Journal of Biostatistics, 3*(1). doi:10.2202/1557-4679.1083 10.2202/1557-4679.1083 22550653

[CR29] Kasper JD, Freedman VA, Spillman BC, Wolff JL (2015). The disproportionate impact of dementia on family and unpaid caregiving to older adults. Health Affairs.

[CR30] Keehan, S. P., Poisal, J. A., Cuckler, G. A., Sisko, A. M., Smith, S. D., Madison, A. J., . . . Lizonitz, J. M. (2016). National health expenditure projections, 2015–25: Economy, prices, and aging expected to shape spending and enrollment. *Health Affairs, 35,* 1522–1531. 10.1377/hlthaff.2016.0459 27411572

[CR31] Langa, K. M., Larson, E. B., Crimmins, E. M., Faul, J. D., Levine, D. A., Kabeto, M. U., & Weir, D. R. (2017). A comparison of the prevalence of dementia in the United States in 2000 and 2012. *JAMA Internal Medicine, 177,* 51–58. 10.1001/jamainternmed.2016.6807 PMC5195883 27893041

[CR32] Langa, K. M., Plassman, B. L., Wallace, R. B., Herzog, A. R., Heeringa, S. G., Ofstedal, M. B., . . . Willis, R. J. (2005). The Aging, Demographics, and Memory Study: Study design and methods. *Neuroepidemiology, 25,* 181–191. 10.1159/000087448 16103729

[CR33] Lees R, Selvarajah J, Fenton C, Pendlebury ST, Langhorne P, Stott DJ, Quinn TJ (2014). Test accuracy of cognitive screening tests for diagnosis of dementia and multidomain cognitive impairment in stroke. Stroke.

[CR34] Leffondré K, Touraine C, Helmer C, Joly P (2013). Interval-censored time-to-event and competing risk with death: Is the illness-death model more accurate than the Cox model?. International Journal of Epidemiology.

[CR35] Lönnroos E, Kyyrönen P, Bell JS, van der Cammen TJM, Hartikainen S (2013). Risk of death among persons with Alzheimer’s disease: A national register-based nested case-control study. Journal of Alzheimer’s Disease.

[CR36] Loomis D, Richardson DB, Elliott L (2005). Poisson regression analysis of ungrouped data. Occupational and Environmental Medicine.

[CR37] Loukine L, Waters C, Choi BCK, Ellison J (2012). Impact of diabetes mellitus on life expectancy and health-adjusted life expectancy in Canada. Population Health Metrics.

[CR38] Meller I, Fichter MM, Schroppel H (1999). Mortality risk in the octo- and nonagenerians: Longitudinal results of an epidemiological follow-up community study. European Archives of Psychiatry and Clinical Neuroscience.

[CR39] Mooney CZ (1997). *Monte Carlo simulation* (Paper Series on Quantitative Applications in the Social Sciences, No. 07-116).

[CR40] Murray, C. J. L., Vos, T., Lozano, R., Naghavi, M., Flaxman, A. D., Michaud, C., . . . Memish, Z. A. (2012). Disability-adjusted life years (DALYs) for 291 diseases and injuries in 21 regions, 1990–2010: A systematic analysis for the Global Burden of Disease Study 2010. *Lancet, 380,* 2197–2223. 10.1016/S0140-6736(12)61689-4 23245608

[CR41] Ostbye T, Hill G, Steenhuis R (1999). Mortality in elderly Canadians with and without dementia: A 5-year follow-up. Neurology.

[CR42] Palloni A, Thomas JR (2013). Estimation of covariate effects with current status data and differential mortality. Demography.

[CR43] Plassman, B. L., Langa, K. M., Fisher, G. G., Heeringa, S. G., Weir, D. R., Ofstedal, M. B., . . . Wallace, R. B. (2007). Prevalence of dementia in the United States: The Aging, Demographics, and Memory Study. *Neuroepidemiology, 29,* 125–132. 10.1159/000109998 PMC2705925 17975326

[CR44] Plassman, B. L., Langa, K. M., McCammon, R. J., Fisher, G. G., Potter, G. G., Burke, J. R., . . . Wallace, R. B. (2011). Incidence of dementia and cognitive impairment, not dementia in the United States. *Annals of Neurology, 70,* 418–426. 10.1002/ana.22362 PMC3139807 21425187

[CR45] Preston, S. H., Heuveline, P., & Guillot, M. (2001). *Demography*. Oxford, UK: Blackwell.

[CR46] Preston, S. H., & Wang, H. (2006). Sex mortality differences in the United States: The role of cohort smoking patterns. *Demography, 43,* 631–646. 10.1353/dem.2006.0037 17236538

[CR47] R Core Team (2014). R: A language and environment for statistical computing.

[CR48] Rhee N (2013). *The retirement savings crisis: Is it worse than we think?* (Report).

[CR49] Ruitenberg A, Ott A, van Swieten JC, Hofman A, Breteler MMB (2001). Incidence of dementia: Does gender make a difference?. Neurobiology of Aging.

[CR50] Salomon JA, Mathers CD, Murray CJL, Ferguson B (2001). *Methods for life expectancy and healthy life expectancy uncertainty analysis* (Global Programme on Evidence for Health Policy Working Paper No. 10).

[CR51] Satizabal CL, Beiser AS, Chouraki V, Chêne G, Dufouil C, Seshadri S (2016). Incidence of dementia over three decades in the Framingham Heart Study. New England Journal of Medicine.

[CR52] Schrijvers EMC, Verhaaren BFJ, Koudstaal PJ, Hofman A, Ikram MA, Breteler MMB (2012). Is dementia incidence declining? Trends in dementia incidence since 1990 in the Rotterdam Study. Neurology.

[CR53] Seshadri S, Wolf PA (2007). Lifetime risk of stroke and dementia: Current concepts, and estimates from the Framingham Study. Lancet Neurology.

[CR54] Seshadri S, Wolf PA, Beiser A, Au R, McNulty K, White R, D’Agostino RB (1997). Lifetime risk of dementia and Alzheimer’s disease: The impact of mortality on risk estimates in the Framingham Study. Neurology.

[CR55] StataCorp. (2015). *Stata Statistical Software: Release 14*. College Station, TX: StataCorp LP.

[CR56] Todd S, Barr S, Roberts M, Passmore AP (2013). Survival in dementia and predictors of mortality: A review. International Journal of Geriatric Psychiatry.

[CR57] Tom, S. E., Hubbard, R. A., Crane, P. K., Haneuse, S. J., Bowen, J., McCormick, W. C., . . . Larson, E. B. (2015). Characterization of dementia and Alzheimer’s disease in an older population: Updated incidence and life expectancy with and without dementia. *American Journal of Public Health, 105,* 408–413. 10.2105/AJPH.2014.301935 PMC4318311 25033130

[CR58] Tschanz, J. T., Corcoran, C., Skoog, I., Khachaturian, A. S., Herrick, J., Hayden, K. M., . . . Cache County Study Group. (2004). Dementia: The leading predictor of death in a defined elderly population—The Cache County Study. *Neurology, 62,* 1156–1162. 10.1212/01.wnl.0000118210.12660.c2 15079016

[CR59] Venables WN, Ripley BD (2002). Modern applied statistics with S.

[CR60] Villarejo, A., Benito-León, J., Trincado, R., Posada, I. J., Puertas-Martín, V., Boix, R., . . . Bermejo-Pareja, F. (2011). Dementia-associated mortality at thirteen years in the NEDICES Cohort Study. *Journal of Alzheimer’s Disease, 26,* 543–551. 10.3233/JAD-2011-110443 21694455

[CR61] Witthaus E, Ott A, Barendregt JJ, Breteler M, Bonneux L (1999). Burden of mortality and morbidity from dementia. Alzheimer Disease and Associated Disorders.

[CR62] Ziegler-Graham K, Brookmeyer R, Johnson E, Arrighi HM (2008). Worldwide variation in the doubling time of Alzheimer’s disease incidence rates. Alzheimer’s & Dementia.

[CR63] Zissimopoulos, J., Crimmins, E., & St.Clair, P. (2015). The value of delaying Alzheimer’s disease onset. *Forum for Health Economics and Policy, 18,* 25–40. 10.1515/fhep-2014-0013 PMC4851168 27134606

