# Supplementary material for: Risk of Developing Dementia at Older Ages in the United States
Source: Demography. 2017 Aug 3;54(5):1897–919. doi: 10.1007/s13524-017-0598-7 (PMC5624986; doi:10.1007/s13524-017-0598-7)
Supplement: Supplementary file 1 — (PDF 665 kb) [file 13524_2017_598_MOESM1_ESM.pdf]

Online Resource 1

Risk of Developing Dementia at Older Ages in the United States

Ezra Fishman

Online supplemental tables: Results using alternative strategy for censoring

Results shown here censor subjects whose last ADAMS status was "alive, dementia-free" at the end of the ADAMS study period, rather than at their last assessment.

Incidence and mortality rate ratios

Age	Estimated Incidence	(SE)	Estimated RR	(SE)
70	0.010	(0.003)	10.56	(4.290)
75	0.015	(0.003)	7.94	(2.322)
80	0.022	(0.003)	6.04	(1.200)
85	0.032	(0.005)	4.67	(0.699)
90	0.046	(0.009)	3.65	(0.664)
95	0.068	(0.019)	2.89	(0.777)
100	0.099	(0.035)	2.33	(0.874)

SE = Standard Error; RR = ratio of morality rates, (with dementia) / (without dementia).

Life table quantities – males – 1920 cohort life table

Age	LE	DFLE	DLE	(SE)	Prob. Demen.	(SE)	DFLE'	(SE)
70	12.31	11.83	0.48	(0.186)	0.252	(0.033)	12.19	(0.150)
75	9.65	9.21	0.44	(0.182)	0.249	(0.033)	9.63	(0.106)
80	7.26	6.85	0.42	(0.163)	0.240	(0.036)	7.28	(0.069)
85	5.20	4.82	0.37	(0.140)	0.225	(0.044)	5.24	(0.042)
90	3.64	3.31	0.34	(0.119)	0.211	(0.054)	3.66	(0.035)
95	2.61	2.28	0.33	(0.112)	0.205	(0.065)	2.59	(0.036)
100	2.02	1.63	0.39	(0.136)	0.216	(0.083)	1.95	(0.047)

Life table quantities – females – 1920 cohort life table

Age	LE	DFLE	DLE	(SE)	Prob. Demen.	(SE)	DFLE'	(SE)
70	15.25	13.84	1.41	(0.249)	0.323	(0.038)	14.27	(0.234)
75	11.91	10.59	1.32	(0.255)	0.312	(0.039)	11.22	(0.157)
80	8.91	7.76	1.14	(0.245)	0.295	(0.044)	8.46	(0.111)
85	6.37	5.45	0.92	(0.220)	0.274	(0.052)	6.10	(0.071)
90	4.42	3.70	0.72	(0.186)	0.252	(0.062)	4.24	(0.042)
95	3.10	2.51	0.59	(0.160)	0.238	(0.073)	2.95	(0.035)
100	2.32	1.74	0.57	(0.164)	0.242	(0.090)	2.18	(0.044)

LE = life expectancy; DFLE = dementia-free life expectancy; DLE = life expectancy with dementia; SE = standard error; Prob. Demen. = probability that a dementia-free person of given age will develop dementia before death; DFLE' = dementia-free life expectancy, conditional on being dementia-free at given age. By design, LE has no standard error and DFLE and DLE have the same standard error.

Life table quantities – males – 1940 cohort life table

Age	LE	DFLE	DLE	(SE)	Prob. Demen.	(SE)	DFLE'	(SE)
70	13.64	12.88	0.76	(0.222)	0.289	(0.036)	13.28	(0.193)
75	10.65	9.95	0.70	(0.223)	0.282	(0.036)	10.47	(0.139)
80	7.96	7.35	0.62	(0.207)	0.270	(0.041)	7.91	(0.097)
85	5.70	5.18	0.52	(0.181)	0.253	(0.050)	5.71	(0.061)
90	4.05	3.59	0.46	(0.156)	0.239	(0.062)	4.05	(0.047)
95	2.95	2.50	0.45	(0.147)	0.235	(0.076)	2.90	(0.049)
100	2.30	1.77	0.52	(0.167)	0.247	(0.096)	2.20	(0.061)

Life table quantities – females – 1940 cohort life table

Age	LE	DFLE	DLE	(SE)	Prob. Demen.	(SE)	DFLE'	(SE)
70	15.99	14.41	1.58	(0.288)	0.349	(0.040)	14.85	(0.269)
75	12.62	11.11	1.51	(0.294)	0.340	(0.043)	11.80	(0.194)
80	9.57	8.22	1.35	(0.285)	0.325	(0.049)	9.01	(0.145)
85	6.93	5.80	1.13	(0.261)	0.304	(0.059)	6.57	(0.098)
90	4.89	3.97	0.91	(0.229)	0.283	(0.070)	4.65	(0.063)
95	3.49	2.71	0.77	(0.202)	0.271	(0.084)	3.29	(0.051)
100	2.63	1.88	0.76	(0.202)	0.276	(0.103)	2.44	(0.058)

LE = life expectancy; DFLE = dementia-free life expectancy; DLE = life expectancy with dementia; SE = standard error; Prob. Demen. = probability that a dementia-free person of given age will develop dementia before death; DFLE' = dementia-free life expectancy, conditional on being dementia-free at given age. By design, LE has no standard error and DFLE and DLE have the same standard error.

Online supplemental table: Incidence of dementia under various intervention scenarios

Mean of 1,000 simulations

New cases of dementia per person-year lived at risk

Age	Scenario 1&2	Scenario 3&4	Scenario 5
70	0.0095	0.00685	0.0058
75	0.0144	0.01032	0.0084
80	0.0220	0.01566	0.0122
85	0.0337	0.02394	0.0178
90	0.0518	0.03675	0.0261
95	0.0794	0.05648	0.0384
100	0.1201	0.08636	0.0565

Scenario 1&2: Delay of dementia onset by 1 year.
Scenario 3&4: Delay of dementia onset by 5 years.
Scenario 5: Reduction in beta-coefficient (slope of incidence increase with age) by 10%.

Online supplemental table: Approximated dementia-free survivors to middle of age interval ($\hat{L}_x^{DF}$) versus dementia-free person-years lived in the interval (L_x^{DF})

Males, deterministic calculation

Age	$\hat{L}_x^{DF}$	L_x^{DF}	Absolute Difference	Diff. as % of L_x^{DF}
71	91351.4	91329.4	22.01	0.02%
72	87440.3	87376.9	63.41	0.07%
73	83361.0	83292.3	68.68	0.08%
74	79139.0	79140.9	1.89	0.00%
75	74991.4	74947.0	44.39	0.06%
76	70708.7	70676.5	32.14	0.05%
77	66374.0	66352.5	21.43	0.03%
78	62007.1	61983.9	23.16	0.04%
79	57592.1	57561.6	30.58	0.05%
80	53108.6	53124.7	16.08	0.03%
81	48703.9	48719.4	15.48	0.03%
82	44329.6	44306.6	23.00	0.05%
83	39870.7	39899.1	28.40	0.07%
84	35520.1	35556.9	36.79	0.10%
85	31251.5	31309.9	58.48	0.19%
86	27121.4	27202.5	81.03	0.30%
87	23176.1	23282.8	106.73	0.46%
88	19469.8	19599.9	130.06	0.66%
89	16047.1	16200.4	153.31	0.95%
90	12954.2	13123.7	169.55	1.29%
91	10216.6	10399.1	182.48	1.75%
92	7857.0	8044.1	187.15	2.33%
93	5876.3	6061.0	184.73	3.05%
94	4262.6	4437.8	175.24	3.95%
95	2990.0	3152.1	162.16	5.14%
96	2028.6	2169.3	140.71	6.49%
97	1327.1	1444.4	117.28	8.12%
98	836.8	930.6	93.80	10.08%
99	510.6	579.7	69.12	11.92%
100	298.0	348.5	50.55	14.51%

The $\hat{L}_x^{DF}$ values were used to estimate the dementia prevalence in each age group using the non-stationary approach and were estimated assuming linearity of survival within one-year age intervals; see Methods section and Appendix for details.
